# Supplementary material for: Clinical responses to adoptive T-cell transfer can be modeled in an autologous immune-humanized mouse model
Source: Nat Commun. 2017 Sep 27;8:707. doi: 10.1038/s41467-017-00786-z (PMC5617838; doi:10.1038/s41467-017-00786-z)
Supplement: Supplementary file 1 — Supplementary Information [file 41467_2017_786_MOESM1_ESM.pdf]

Description of Supplementary Files

File Name: Supplementary Information
Description: Supplementary Figures

File Name: Peer Review File
Description:

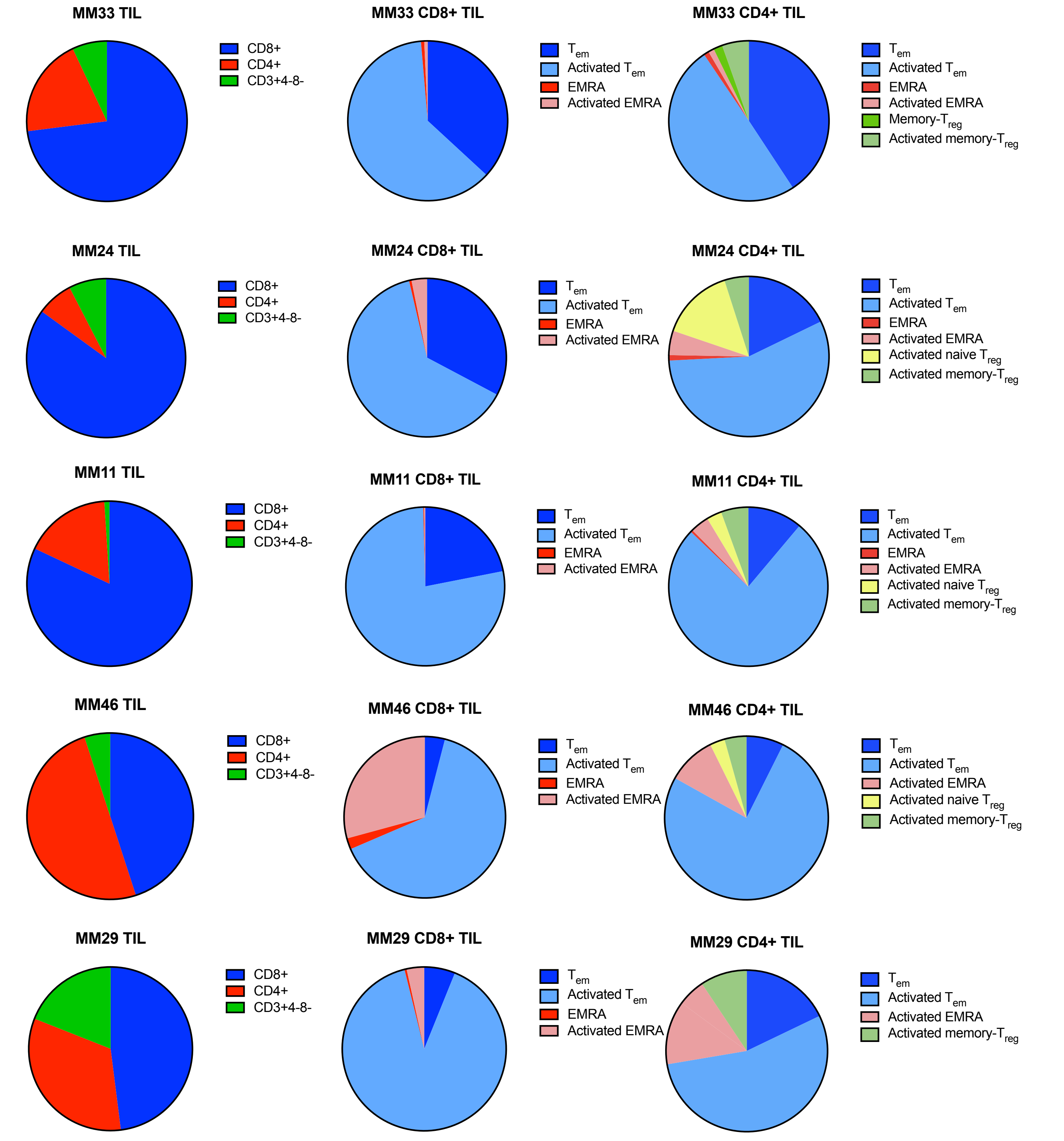

Supplementary Figure 1. Detailed immune phenotype of TILs used in PDX experiments. CD8+ cells is the dominating cell type in TILs expanded through REP. The CD8+ population consists of effector memory cells (and effector memory RA (EMRA)), the vast majority expressing HLA-DR (activated). In the CD4+ population a small proportion of regulatory T cells (T-reg) could be detected.

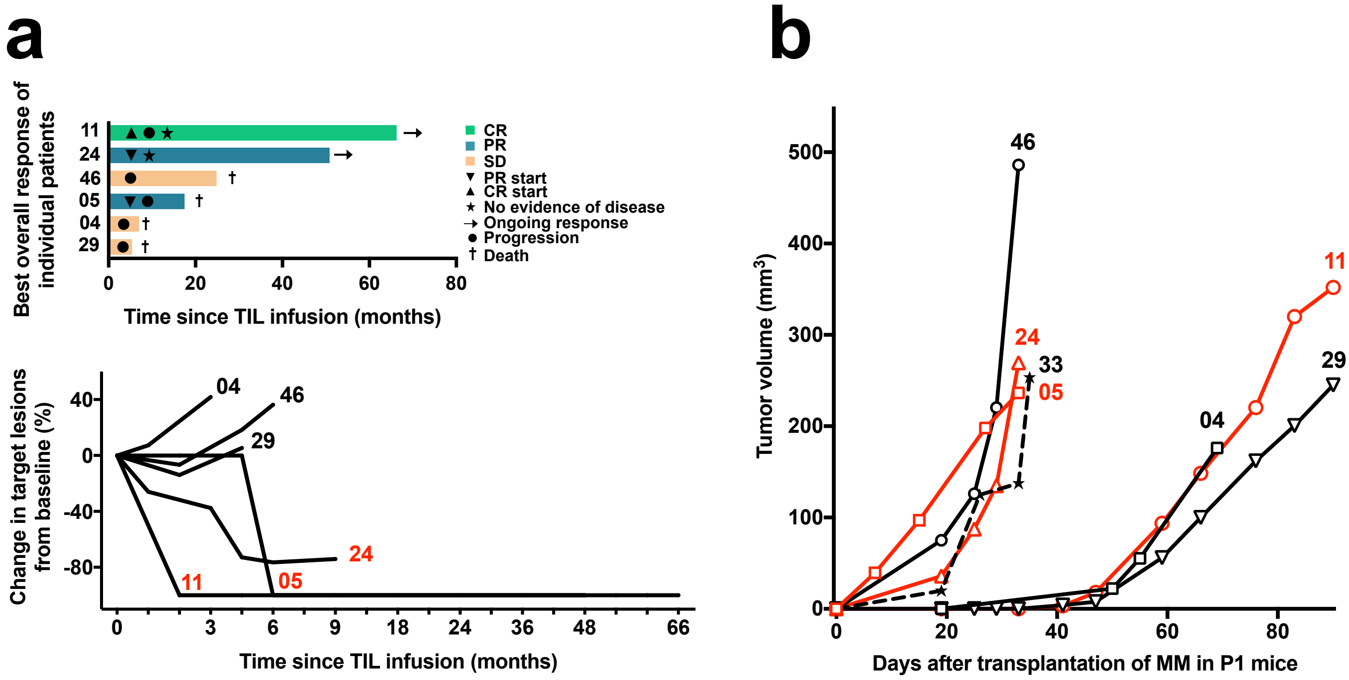

Supplementary Figure 2. Clinical outcome of patients from which the tumors and TILs come and tumor growth in mice.

a) Responses of indicated patients to adoptive cell therapy. Shown are time to and duration of response (swimmer plot, upper panel) and change in target lesions (spider plot, lower panel). b) First passage tumor growth curves in NOG mice of melanoma cells from indicated patients (n=1 per sample).

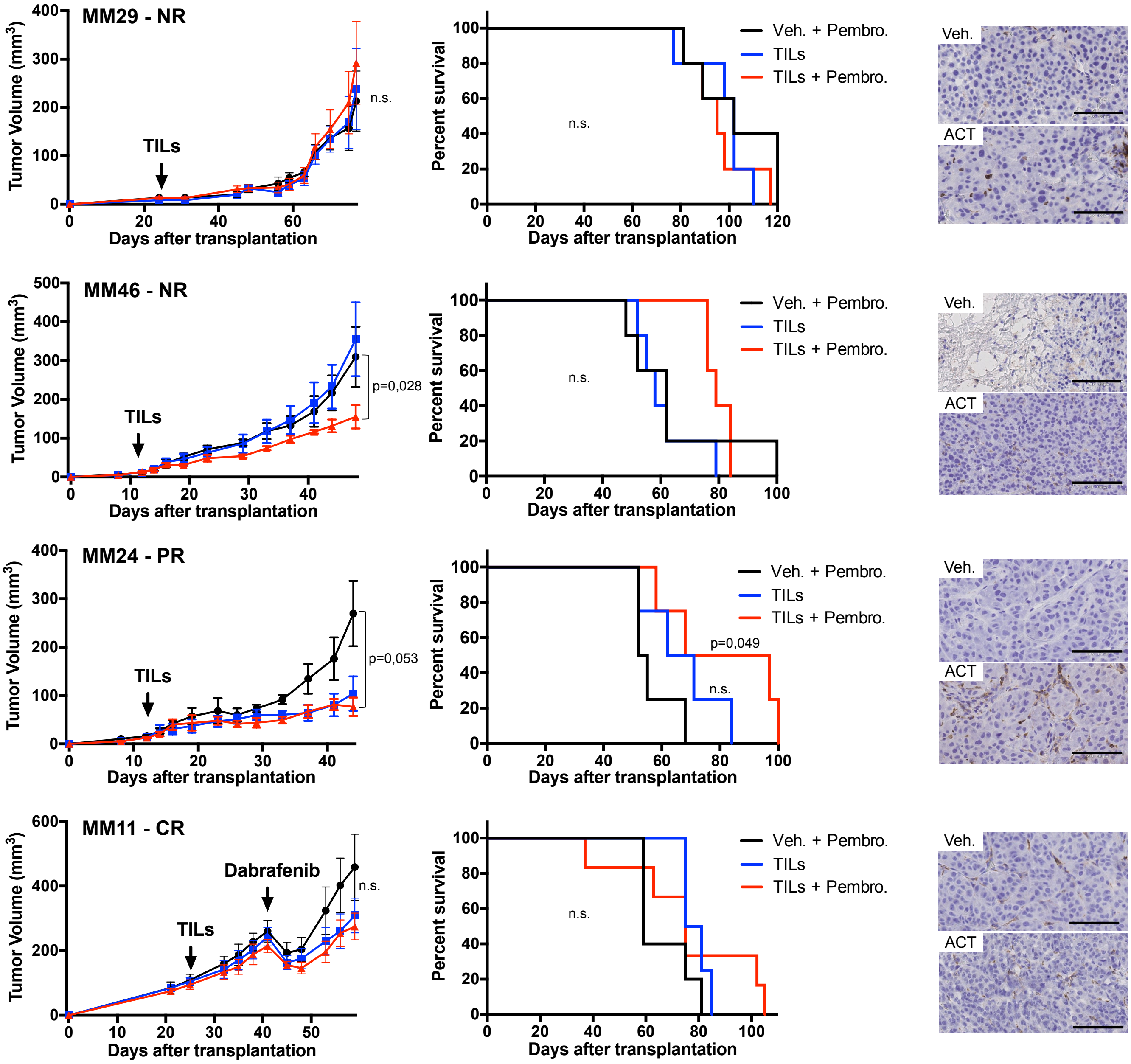

Supplementary Figure 3. Adoptive T cell transfer in NOG mice does not cause tumor rejection irrespective of clinical outcome of patients from which the tumors and TILs come. Tumor growth curves and corresponding survival curves (based on ethical limits) of NOG mice transplanted with cells from indicated patients either injected with autologous TILs and/or treated with anti-PD1 antibody pembroluzimab twice weekly (n=4 per group for MM24, n=5 per group in all other samples). 45000 IU IL-2 (clinical grade Aldesleukin, Novartis) was given daily for three days following TIL injection and thereafter twice weekly. Right panel contains tumor immunohistochemistry (CD3 staining in the first ACT and control tumors reaching the ethical limit, bar=100µm).

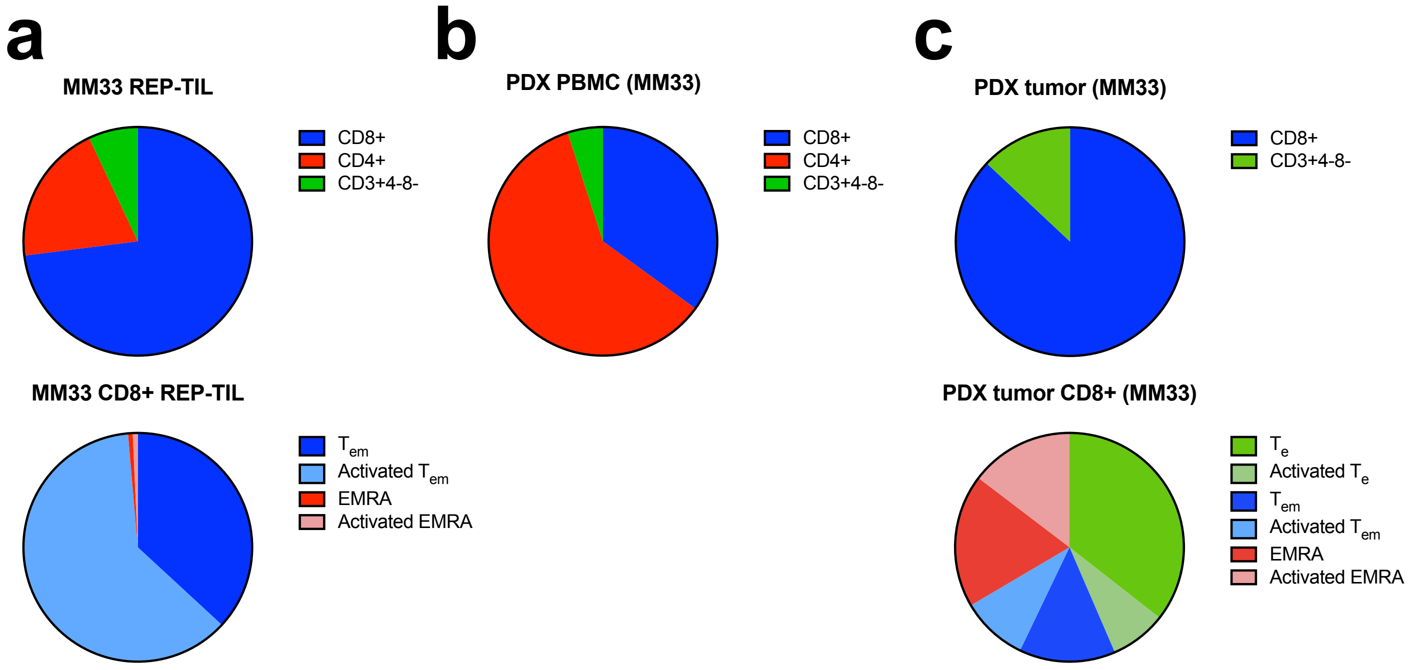

Supplementary Figure 4. TILs can be detected by flow cytometry in blood and tumor from mice treated with ACT. a-c) Comparison of TIL phenotypes in infusion product (REP-TILs) (a), blood (b) and tumor homogenate (c) from transgenic *hIL2*-NOG mice transplanted with MM33 tumor cells and treated with autologous TILs.

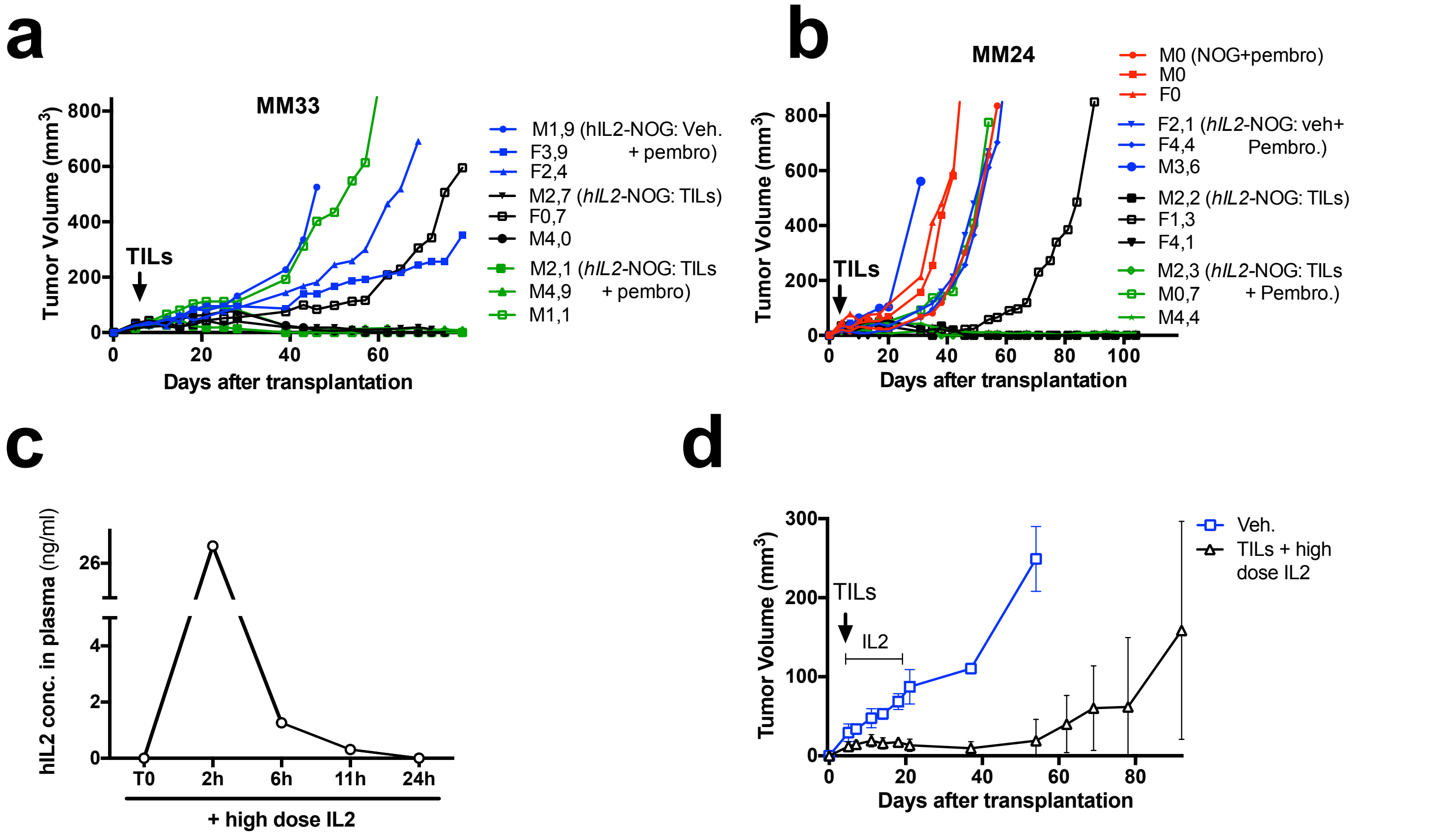

Supplementary Figure 5. **Response to ACT relies on the endogenous level of IL-2 in *hIL2*-NOG mice or on the dose of IL-2 injected to NOG mice.** Individual tumor growth curves of MM33 (a) and MM24 (b) cells transplanted into *hIL2*-NOG mice and then treated with TILs and/or pembrolizumab. Shown in legend are also gender of mouse (F or M) and levels of plasma IL-2 levels (ng/ml). Note the lack of response to TIL therapy in mice expressing less than 1,3 ng/ml. c) Changes in IL-2 plasma levels in NOG mice injected with recombinant IL-2 (2,75µg; > 45000 IU, PeproTech) over time. d) MM33 cells were transplanted into six NOG mice. Five days after transplantation, mice were randomized into two groups, one of which received a tail vein injection with autologous TILs and daily high-dose IL-2 for 16 consecutive days. Tumor growth was measured using calipers.

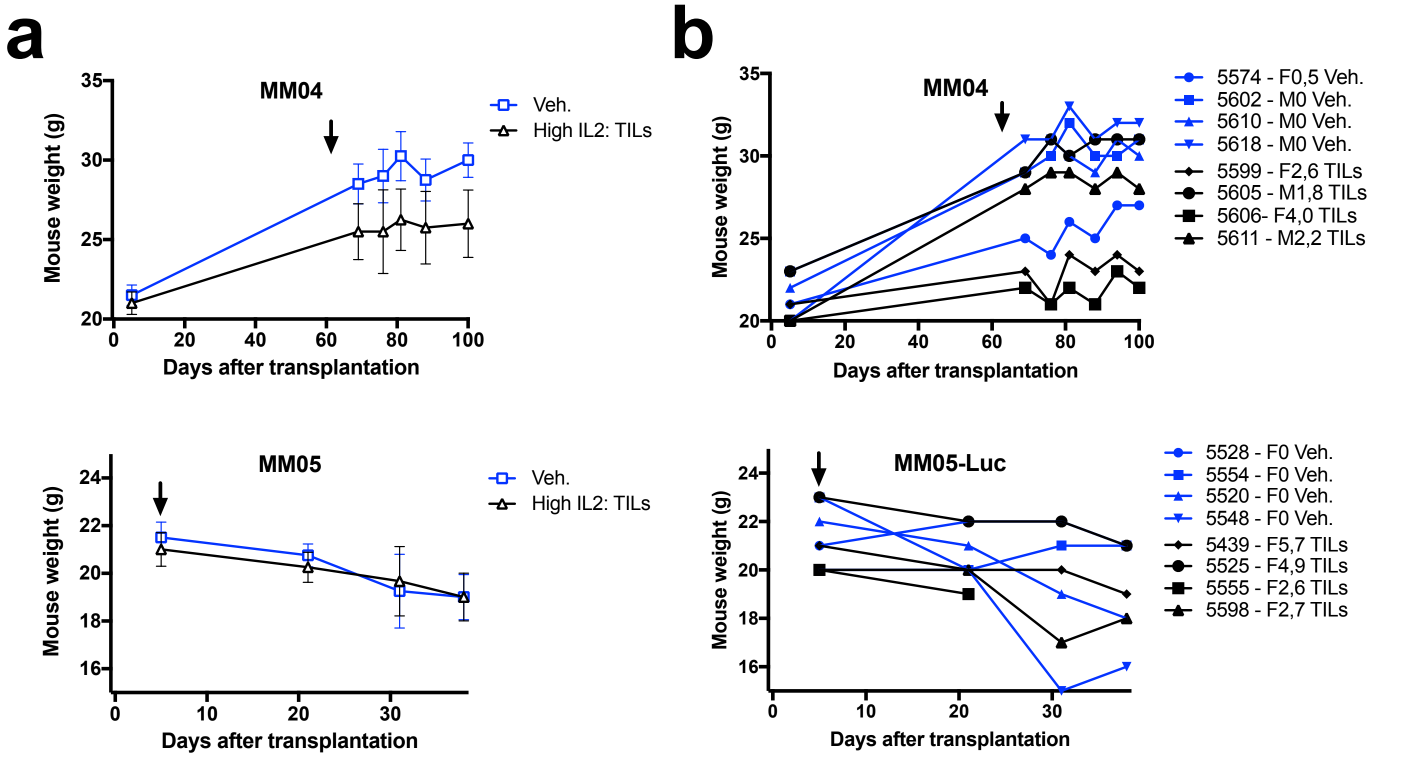

Supplementary Figure 6. ACT does not induce a loss of weight in *hIL2*-NOG mice that respond or not to the treatment. Mean (a) and individual (b) weights of mice bearing MM04 and MM05 and treated with TILs.
